# Effects of incorporating agro-industrial by-products into diet of New Zealand rabbits: Case of rebus of date and apricot kernel meal

**DOI:** 10.14202/vetworld.2017.1456-1463

**Published:** 2017-12-12

**Authors:** Achour Mennani, Rafik Arbouche, Yasmine Arbouche, Etienne Montaigne, Fodil Arbouche, Halima Saâdia Arbouche

**Affiliations:** 1 Department of Agronomy, Faculty of Science of Nature and Life, University of El Tarf, Algeria; 2 Department of Agronomy, Faculty of Science of Nature and Life, University of Ghardaia, Algeria; 3 Department of Agronomy, Faculty of Science of Nature and Life, University of Setif, Algeria; 4 Joint Research Unit “Market, organization, institution, actors strategies, University Supagro of Montpellier, France

**Keywords:** agro-industrial by-product, apricot kernel meal, fattening, rabbits, rebus of dates

## Abstract

**Aim:**

The aim of this study was to determine the effects of incorporating the by-products complex of date and apricot on the fattening performance of the New Zealand breed of rabbits, to reduce the economic costs of the food formula.

**Materials and Methods:**

A total of 288 young New Zealand rabbits aged 35 days were divided into four equal groups each containing 72 animals and into sub-groups of 6 rabbits per cage, depending on the rate of substitution of corn by date rebus and of soybean meal by apricot kernel meal (0%, 10%, 20%, and 30%).

**Results:**

The change in weight from day 35 to 77 and the average daily gain are not significantly different, regardless of the diet. The pH and water content are proportional to the substitution rates (6.4-6.6% and 66.5-68.8%). Meat protein levels increased significantly, in particular for the 10% and 30% groups (+8.1% and 6%) while the fat and mineral content levels decreased significantly, in particular for the 30% group displaying −16% and −17%, respectively. Incorporation of dates and apricot kernel meal into the ration of rabbits reduces the cost of the kilogram of food produced of −9%, with an opportunity cost of 165 Algerian dinars (DZD).

**Conclusion:**

The date rebus/apricot kernel meal complex can be used as an alternative to the corn/soybean meal complex at substitution rates of up to 30% without adverse effects on growth rates, feed contribution, or slaughter yield. It improves the chemical composition of the meat and reduces the cost price of the quintal of feed produced.

## Introduction

The breed of rabbits imported by Algeria is primarily the New Zealand breed. These rabbits were imported with a view to improving the local breed and ensuring both a qualitative and quantitative diversification of the meat. In stock farming, spending on food accounts for between 60% and 70% of production costs [1] and this is also true for rabbits (60%) [2]. The use of unconventional foodstuffs is one of the alternatives that can be adopted to reduce production costs [3-5]. Economically speaking, it would provide the poorer strata of the population with cheap access to animal proteins.

The feed rations of rabbits during the fattening stage essentially consist of soybean meal and corn, raw materials that are imported by Algeria in their entirety. Their use, in the feed ration, can be reduced by substituting date rebus for corn and apricot kernel meal for soybean meal.

The diet of rabbits comprises a single pelleted ration and the impact of replacing corn by date rebus is clearer due to a high rate of crude fiber (24%), which is essential to a rabbit’s digestive transit [6,7] and helps avoid potential outbreaks of enteritis, which can prove fatal [8,9].

The by-products of the date palm contain little protein (5.2% of total proteins), which is concentrated in the stone [10,11]. The chemical composition of these products varies according to the region, the nature of the cultivar (Deglet noor, Mech deglat, Litima) [12] and the proportion of stones in the mixture [13]. On average, they account for 25% of annual date production [14].

Using apricot kernel meal as a source of protein in the diets of domestic animals has only been studied among broiler chickens [15], and sheep used for fertilizer [16] and is thus not a widespread practice. Nevertheless, several studies have examined the hydrocyanic acid content of apricot kernels [17]. This rate depends on the variety of apricot grown (sweet or bitter kernels) [18] and the percentage of each variety in the overall quota handled by the agri-food industry. An increase in the land dedicated to apricot trees in Algeria, made possible by successive National Agricultural Development Plans in particular in the Hrodna region, has led to an accumulation of by-products generated by the agro-industry through production from the young orchards which cover an average area of 47,000 ha [14], planted extensively with a density of 300 trees per hectare.

The aim of this study is to determine the effects of incorporating both “date rebus” and “apricot kernel meal” in the diet of the imported breed (New Zealand) during the fattening phase of young rabbits weaned at 35 days.

## Materials and Methods

### Ethical approval

The present study was conducted after approval of Institutional Animal Ethics Committee laboratory of Agriculture Department of Ghardaia University, Algeria.

### Animals, diets, and experimental protocol

The test was conducted during the period running from 12/03/2015 to 23/04/2015 in the hutches of a professional breeder located in the municipality of Rasfa in the wilaya of Sétif in north-eastern Algeria. Covering an area of 80 m², the building is fitted with heat insulation in the form of polystyrene panels. Fans and a pad-cooling humidifier ensure that the correct atmospheric conditions are maintained.

288 young New Zealand rabbits of both sexes, weaned at the age of 35 days, were randomly assigned to 4 groups of 72 subjects. The rabbits in each group were housed together in metal cages with 6 rabbits/cage, i.e., 12 allocations per group.

The date rebus was provided by the date processing and packaging unit in Tolga, in the wilaya of Biskra, and contained a similar proportion of stones (45%) and fleshes (55%). The whole was dried in the sun then crushed.

The apricot stones (mixture of sweet and bitter stones) were provided by the apricot processing unit located in N’gaous, wilaya of Batna. They were shelled, with the shell and the kernel being separated by hand. After being dried in the sun, the kernel was treated to obtain a meal by means of a hydraulic press, using the principle recommended by Ferradji *et al*. [19], and detoxified using a 1% solution of bicarbonate of soda [18].

The chemical composition of the date rebus and apricot kernel meal (Table-1) was determined using the methods of the AOAC [20], repeated 3 times. The analyses examined the dry matter, total nitrogenous matter, crude fiber, fat, mineral content, and hydrocyanic acid; the gross energy was determined by adiabatic calorimetry and the amino acids by high-performance liquid chromatography.

**Table-1:** Chemical composition of the date rebus and apricot kernel meal.

Elementary composition	Date rebus	Apricot kernel meal
Organic latter (% DM)	94	96.70
Total nitrogenous matter (% DM)	5	42.30
Crude fiber (% de DM)	24	7.7
Fat (% DM)	7	10.4
Mineral content (% DM)	6	3.3
Nitrogen-free extract (% DM)	58	36.7
HCN (mg/100g DM)	0	102
NDF (% de MS)	40.2	18.4
ADF (% de MS)	32.3	10.7
ADL (% de MS)	4.6	7.4
Hémicellulose (% de MS)	13.6	7.7
Gross energy (kcal/kg DM)	4235	5180
Digestible rabbit energy (kcal/kg DM)*	3152	3984
Digestible rabbit protein (g/kg of DM)^#^	9.1	336
Lysine (g/100 g of foodstuff)	3.2	1.8
Methionine (g/100 g of foodstuff)	1.5	1.2
Cystine (g/100 g of foodstuff)	1.7	1.3

DM=Dry matter, NDF=Neutral detergent fiber, ADF=Acid detergent fiber, ADL=Acid detergent lignin.

* Estimated by the equation of Maertens *et al*. (1988): DRE (kcal/kg DM)=0.8-0.230 ADF (%DM) + 0.80 GE (kcal/kg DM),

# Estimated by the equation of Villamide and Fraga (1998): DRP (g/kg)=−34.67+0.876×TNM (g/kg)

Four rations were formulated with WAFFDA [21] software containing 0% (control feed), 10%, 20%, and 30% kernel meal replacing the soybean meal and data rebus replacing the corn during the fattening phase (Table-2). The pelleted feed was distributed at will every day at 9 a.m. and 4 p.m.

**Table-2:** Formula (kg/100 kg of feed) of the feed distributed to the rabbits according to the substitution rates of soybean meal by apricot kernel meal and of corn by date rebus.

% Substitution	0%	10%	20%	30%
Ingredients				
Corn	20	18	16	14
Date rebus	0	2	4	6
Soybean meal	12.7	11.43	10.16	8.89
Apricot kernel meal	0	1.27	2.54	3.81
Wheat bran	32	32	32	32
Wheat straw	4.7	4.7	4.7	4.7
Dried alfalfa	29	29	29	29
Salt (NaCl)	0.5	0.5	0.5	0.5
Sel (Nacl)	0.5	0.5	0.5	0.5
Rabbit premix (CMV)	0.5	0.5	0.5	0.5
Calcium carbonate	0.5	0.5	0.5	0.5
L-Lysine	0.085	0.085	0.085	0.085
DL-Methionine	0.015	0.015	0.015	0.015
Content of calculated nutrients				
Crude fiber (%)	15.20	15.64	16.08	16.52
NDF (%)	33.71	34.07	34.44	34.80
ADF (%)	18.72	18.96	19.20	19.44
ADL (%)	4.24	4.40	4.57	4.73
Hemicellulose (%)	14.99	15.11	15.23	15.36
Lysine (%)	0.82	0.85	0.89	0.92
Methionine (%)	0.24	0.28	0.31	0.34
Total sulfur amino acids (%)	0.50	0.52	0.55	0.57
Digestible proteins (%)	11.16	11.05	10.93	10.82
Digestible rabbit energy (kcal/kg)	2483	2473	2463	2453
Metabolizable rabbit energy (kcal/kg)	2373	2369	2365	2361
Cellulose versus ADF-ADL (%)	14.48	14.56	14.63	14.71
PD/ED calculated g/1000 kcal	44.95	44.67	44.39	44.11

Premix (rabbit CMV at 1%) provided per kg diet: Se=0.08, Mg=2.6, Mn=2.0, Zn=6.0, I=0.08, Fe=4.0, Cu=1.10, S=6.8, Co=0.04, Thiamin=0.20, Riboflavin=0.20, Calcium d-pantothenate=0.8, Pyridoxine=0.10, Biotin=0.004, Nicotinic acid=2, Choline chloride=12, Folic acid=0.20, Vitamin K3=0.1, dl-α-tocopheryl acetate=2.0, Folic acid=0.2, Cyanocobalamin=0.002, Vitamin A=950000 IU, Vitamin D3=120000 IU, NDF=Neutral detergent fiber, ADF=Acid detergent fiber, ADL=Acid detergent lignin.

The animals were weighed individually at 35, 49, 63, and 77 days at an interval of 15 days with a diet day before weighing. The food consumption of each cage was checked every week of the experiment at a specific time.

The slaughter parameters and carcass characteristics were determined using the methods proposed by Blasco *et al*. [22], Blasco and Ouhayoun [23], Ouhayoun and Dalle [24], and Blasco and Gomez [25]. They were based on body weight at slaughter (bws), hot carcass weight (hcw), cold carcass weight (ccw), reference carcass weight (rcw), hot carcass yield (hcw/bws), cold carcass yield (ccw/bws), weight of liver (wl), wl/bws ratio, weight of perirenal fat (wprf), perirenal fat/body weight ratio (wprf/bws), perirenal fat/hot carcass ratio (wprf/hcw), weight of skin (ws), ws/bws ratio, weight of digestive tract full, weight of digestive tract full/body weight at slaughter ratio (wdrf/bws), weight of front section (wfs), wfs/bcw ratio, weight of rear section (wrs), wrs/bcw ratio, weight of intermediate “saddle” section (wiss), and wiss/bcw ratio. The pH of the *Longissimus lumborum* muscle was measured in the meat directly 1 h postmortem using a pH-meter. The chemical composition of the meat was determined using the AOAC methods with the sampling repeated 3 times. The analyses examined the water content, proteins, fat, and ashes.

### Statistical analysis

The different results were processed using the Microsoft Excel spreadsheet. The statistical analysis and comparison of averages between the different dietary schemes (control and those based on apricot kernel cake and date rebus) were conducted by means of the one-way analysis of variance (ANOVA) test using the Statistical Package for the Social Sciences software (SPSS version 21), then completed by means of the Student-Newman-Keuls and Duncan test if the ANOVA test displayed a significant difference from the error risk of 5% (p<0.05).

### Economic analysis

An initial estimation of the economic benefit of replacing the foodstuffs traditionally used to formulate the feed given to young New Zealand rabbits, corn and soybean, with the by-products date rebus and apricot kernel meal can be conducted by evaluating the value of the compounds replaced.

By considering that this incorporation-substitution was implemented at the same operational cost (not including material costs) as that of the corn and soybean meal, this value serves to remunerate the activities associated with using these by-products. Only the direct variable costs are thus taken into account here.

The calculation, therefore, involves evaluating the savings made by replacing corn and soybean in the ration. In our case, we perform two replacements and assume that the price of date rebus is fixed. The opportunity cost of apricot kernel cake is thus the unit price for which the total cost of the ration would remain unchanged. As the weight gain is almost identical in all four groups, we can calculate the opportunity cost of this cake by comparing the quantity used to the unit price. In our case, this is DZD 55/kg.

- In accordance with Table-3: for the 10% ration

**Table-3:** Change in slaughter parameters and carcass characteristics of young New Zealand rabbits during the fattening phase according to the percentage substitution of soybean meal by apricot kernel meal and of corn by date rebus.

Slaughter parameters	0%	10%	20%	30%	SEM	p
Body weight at slaughter (bws) (g)	1886	1979	1927	1996	24.39	NS
Hot carcass weight (hcw) (g)	1484	1475	1479	1455	7.09	NS
Cold carcass weight (ccw) (g)	1191	1255	1271	1256	15.35	NS
Reference carcass weight (rcw) (g)	916	966	987	979	12.45	NS
hcw/bws yield (%)	78.7	74.7	76.7	72.9	0.77	NS
ccw/bws yield (%)	63.2	63.5	65.9	62.9	0.59	NS
Muscle/bone ratio	8.05	8.04	8.37	8.76	0.16	NS
Carcass characteristics						
Weight of liver (wl) (g)	52.6	57.3	56.6	62	2.09	NS
wl/bws ratio (%)	2.79	2.87	2.94	3.25	0.09	NS
Weight of perirenal fat (wprf) (g)	18.6	22.6	20.6	18.0	1.75	NS
Perirenal fat/body weight ratio (wprf/bws) (%)	0.63	1.14	1.06	0.94	0.08	NS
Perirenal fat/hot carcass ratio (%) (wprf/hcw)	0.72	1.79	1.62	1.42	0.13	NS
Weight of skin (ws) (g)	192	195	191	211	4.26	NS
ws/bws ratio (%)	10.2	9.8	9.9	11.07	0.19	NS
Weight of digestive tract full (wdrf) (g)	299	303	265	287	9.88	NS
wdrf/bws ratio (%)	15.8	15.2	13.7	15.0	0.43	NS
Weight of front section (wfs) (g)	333^a^	356^b^	371^b^	359^b^	4.63	*
Weight of rear section (wrs) (g)	388	382	392	388	4.86	NS
Weight of intermediate “saddle” section (wiss) (g)	211	227	223	232	6.12	NS
wfs/bcw ratio (%)	17.6	18.0	19.3	18.8	0.29	NS
wrs/bcw ratio (%)	20.6	19.4	20.4	20.4	0.33	NS
wiss/bcw ratio (%)	11.2	11.5	11.6	12.1	0.23	NS
Chemical composition of the meat						
pH	6.41^c^	6.45^a^	6.52^d^	6.58^b^	0.04	***
Water content (%)	66.48^b^	66.00^a^	68.90^d^	67.85^c^	0.34	***
Proteins (%)	19.15^a^	20.70^b^	18.89^d^	20.29^e^	0.23	***
Fat (%)	12.90^a^	11.12^c^	10.71^b^	10.89^d^	0.26	***
Mineral content (%)	1.10^a^	1.00^c^	1.10^a^	0.91^d^	0.02	***

SEM=Standard error of the mean, NS=Not significant, *Significant, *** highly significant. The different exponents indicate significant differences (p<0.05).
- Price of corn: DZD 27/kg
- Price of date rebus: DZD 10/kg
- Quantity of corn saved: 2 kg
- Quantity of dates used: 2 kg
- Savings on corn: DZD 54
- Cost of date rebus: DZD 20
- Corn-date savings: DZD 34
- Price of soybean: DZD 55/kg
- Quantity of soybean saved: 1.27 kg
- Savings on soybean: DZD 70
- Total savings on the new ration DZD 70+DZD 34=DZD 104
- Opportunity cost of the apricot kernel cake=70/1.27=DZD 55

The opportunity cost is the same for the other rations, taking the proportionality of the results into account. This cost can represent or cover (1) the extraction and processing cost of apricot stones, (2) the processing specific to the by-product, extraction of the kernel and processing of the cake, possibly in a specific unit. The intermediate prices of bagged apricots serve to define the costs and benefits of each operator in the value chain. The valuation level of other coproducts, such as apricot kernel oil intended for the pharmaceutical and cosmetic industries, is also involved in distributing this value along the value chain. A detailed analysis of the operators, their cost structure, the market for these by-products and their coproducts goes beyond the scope of this paper.

## Results

During the entire experiment, the mortality rate was low (6.10%) and primarily the result of accidents when handling the animals (weighing).

### Growth, consumption index, intake and average gain

**Figure-1:**
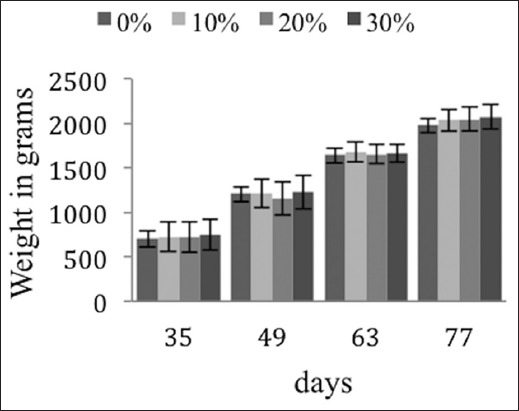
Effects on growth of the New Zealand breed according to the substitution rates. The lack of exponents indicates non-significant (p>0.05) differences among the substitution rate groups.

**Figure-2:**
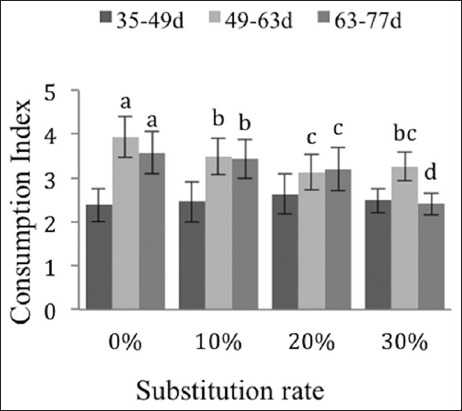
Effects of substitutions on the consumption of the New Zealand breed. The different exponents indicate significant differences (p<0.05).

The development of body weight is not affected by the rates of substitution of corn by date rebus and soybean meal by apricot kernel meal (p>0.05) (Figure-1). During the rearing phase between day 35 and 49, the consumption index does not vary between any of the diets (p>0.05). The regression of the consumption index is less pronounced during the phase from day 49 to 63 for the 20% and 30% groups (−21% and −18%) in relation to the control group. The consumption index falls in proportion to the substitution rates (−4%, −11%, and −33%, respectively) during the rearing phase from day 63 to 77 (Figure-2).

**Figure-3:**
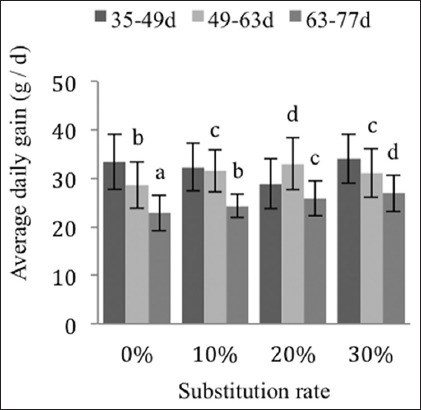
Effects of substitutions on the average daily intake of the New Zealand breed. The different exponents indicate significant differences (p<0.05).

**Figure-4:**
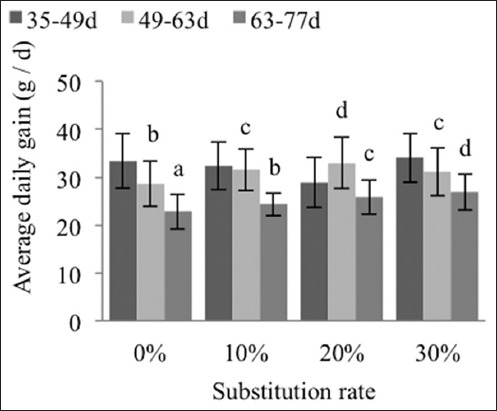
Effects of substitutions on the average daily weight gain of the New Zealand breed. The different exponents indicate significant differences (p<0.05) among the substitution rate groups.

Between the control group and the 30% group, the average daily intake follows the same trend for the rearing phases from day 49 to 63 and from day 63 to 77 (−18% and −17%, respectively) (Figure-3), whereas the average daily gain (ADG) for the rearing phases from day 49 to 63 is less valued than that for the phase from day 63 to 77 with a difference of +10% and +18%, respectively (Figure-4).

**Figure-5:**
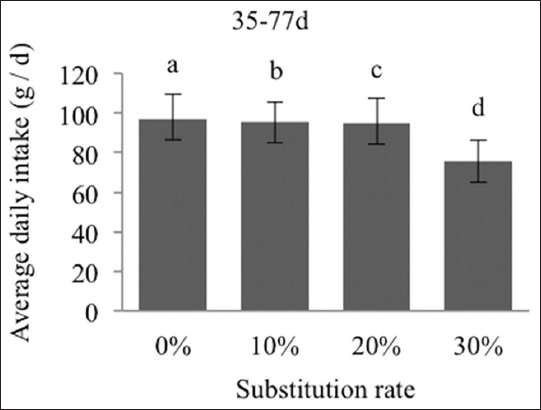
Effects of substitutions on the average daily intake of the New Zealand breed during the overall rearing phase (day 35-77). The different exponents indicate significant differences (p<0.05).

**Figure-6:**
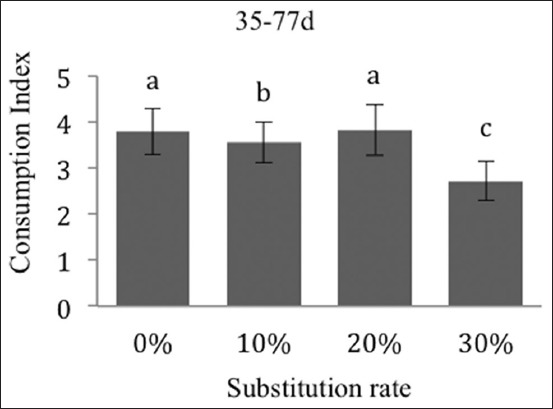
Effects on the consumption index of the New Zealand breed during the overall rearing phase (day 35-77). The different exponents indicate significant differences (p<0.05).

**Figure-7:**
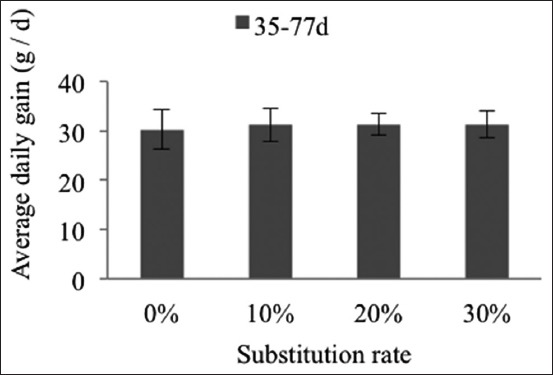
Change in average daily gain of the New Zealand breed during the overall rearing phase (day 35-77). The lack of exponents indicates non-significant differences (p>0.05).

The average daily intake and the consumption index are more expressive for the 30% group, at −23% (Figure-5) and −28% (Figure-6) from the day 35 to 77. The ADG during this period is in no way affected by the substitution rates (Figure-7).

### Slaughter parameters, carcass characteristics and chemical composition of the meat

None of the indicators related to the slaughter parameters and to the carcass characteristics depends significantly on the substitution rate (Table-3) (p>0.05), except the weight of the front section which remains dependent on the rate of incorporation with a more marked influence for the 20% group (+11.4%). The proportional change in the PH of the meat is linked to the substitution rates, with +2.6% for the 30% group. The protein rate increases significantly, with a predominance of the 10% group (+8.1%). The increase in water content is especially marked for the 20% and 30% experimental groups (+3.6% and +2.1%, respectively).

A sharp fall in both fat and mineral content is observed for the 30% group (−16% and −17%, respectively).

### Economic analysis

The production costs of the two by-products were calculated according to either the fixed or variable costs. The apricot kernel meal is obtained by processing the apricot kernels in several stages: Drying, shelling, sorting, extraction of the oil, and detoxification. The two raw materials obtained that can be commercialized the kernel meal and kernel oil. It would be possible to deem that the high level of recovery of the oils would justify, taking into account all costs and sales of latters, a price per kilogram of the kernel meal produced at DZD 00.

The date rebus is generated by the processing and packaging units, and this by-product is provided at a cost of DZD 10/kg.

Taking account of the four formulae used in our study (the control feed with 0% substitution (standard diet), the experimental diets with 10%, 20%, and 30% substitution of soybean meal by apricot kernel meal and of corn by date rebus and the prices adopted on the domestic market (Table-4), the opportunity cost of incorporating the by-products is quite high, at −9% on the price of a quintal of feed produced for an incorporation rate of 30%.

**Table-4:** Price of rabbit concentrates according to the incorporation rates of apricot kernel meal and date rebus.

Raw materials	Price DZD/kg	Control (0%) (kg/q)	Substitution rate (%) (kg/q)		

			10	20	30
Corn	27	20.00	18	16	14
Date rebus	10	0.0	2	4	6
Dried alfalfa	36	29	29	29	29
Kernel cake	00	0.00	1.27	2.54	3.81
Soybean meal 46	55	12.70	11.43	10.16	8.89
Wheat straw	20	4.70	4.70	4.70	4.70
Wheat bran	27	32	32	32	32
Calcium carbonates	1.7	0.5	0.5	0.5	0.5
Salt	30	0.5	0.5	0.5	0.5
CMV rabbit	150	0.5	0.5	0.5	0.5
Methionine	400	0.015	0.015	0.015	0.015
DLlysine	400	0.081	0.085	0.085	0.085
Total		100	100	100	100
Price of rabbit concentrate (DZD/q)		3370	3266	3162	3058
Difference			−3%	−6%	−9%
Difference (DZD/q)			104	208	312

## Discussion

While the initial ADG (day 35-49) does not differ between diets, due to the low correlation between the weight at weaning and the speed of growth [26], the variations in the ADG from day 49 to 77 (p>0.01 and 0.001), seem to be linked to the increasing threshold of the rates of sulfur amino acids in the diets, as shown by Colin [27] (0.63%), Cheeke [28] (0.45%), and Berchiche and Lebas [29] (0.47-0.62%), which would be better assimilated with age.

The ADG for the overall rearing period (day 35-77) does not change. The New Zealand breed seems to digest the fiber in the date rebus and apricot kernel meal very well although, according to Gidenne and Perez [30], the digestibility of fiber may increase or decrease between weaning while the age of slaughter and the source of fiber [31] and the nature of the parietal constituents [32] also play an essential role. The average daily weight gains among the New Zealand rabbits on the experimental and control diets (31.3 vs. 30.2 g) do not achieve the ADG proposed by Szendro and Dalle [33] (40-46 g/d) for the same breed.

Growth at days 49, 63, and 77 is not affected by any of the incorporation rates of the apricot kernel cake or date rebus in the diets (Table-3) and is also dependent on weight at weaning, which is linked to the mothers’ diets [24] and the breed itself [34].

The consumption indices and average daily intakes between days 49 and 77 are significantly different and decrease in proportion to the incorporation rates of the by-products (Figures-2 and 3). This could be due to the polysaccharides contained in the date rebus and to the interaction of the fiber from the date stones and from the kernel meal, as indicated by De Blas and Carabano [31] for various agro-industrial by-products used in rabbit feed.

Incorporating apricot kernel meal and date rebus instead of soybean meal and corn has no effect on the carcass characteristics and slaughter parameters, which could be explained by the low substitution rate of the two by-products, with a yield at slaughter (ccw/bws) of 66%, which remains below the range proposed by Berchiche and Lebas [29] (58-60%).

The weight of the front section is improved by the substitution rate of corn by date rebus and of soybean meal by apricot kernel meal, as well as by the removal of the rib cage and of the trachea, heart and lungs, as shown by Ouyed [34].

The rabbit meat pH results remain different from those of other authors [35,36] and are not affected by the genetic type as proposed by Lambertini *et al*. [37].

They change in proportion to the incorporation rates of date and apricot kernel by-products and remain high (6.60) due to the fact that taking the measurement one hour postmortem does not allow sufficient time for the different glycolytic activities to be triggered in the muscles, as shown by Lambertini *et al*. [36], as this only occurs some 24 h after death. This high pH results in unsuitability for refrigeration as the proteolytic micro-organisms develop bad smells in this case [38].

Due to its hardiness, the local breed draws greater benefit from the protein sources in the diets by means of better protein synthesis and remain within the standards proposed by Ouhayoun [39] (20-23%).

The lipid content of the meat of both breeds is inversely proportional to the incorporation rates of date rebus and apricot kernel meal and is higher in the New Zealand breed. According to Dalle [40], it depends on the age, gender, genotype, feed, and rearing method. It remains within the interval proposed by Salvini *et al*. [41] (0.6-14.4%).

## Conclusion

The date rebus/apricot kernel meal complex can be seen as a rich source of total nitrogenous matter (47%) and fibers (32%) and can be used as an alternative to the corn/soybean meal complex at substitution rates of up to 30% without adverse effects on growth rates, feed contribution or slaughter yield. It improves the conversion rate of feed as well as the chemical composition of the meat and reduces the cost price of the quintal of feed produced. It would be beneficial to increase the substitution rates to determine the optimum thresholds.

## Authors’ Contributions

AM prepared the ground conditions and collected the data. RA revised the manuscript. YA performed the analysis of the data. EM carried out and drafted the economic analysis. FA designed the study and drafted. HSA revised the manuscript. All authors have read and approved the final manuscript.
